# Behavioral factors to include in guidelines for lifelong oral healthiness: an observational study in Japanese adults

**DOI:** 10.1186/1472-6831-6-15

**Published:** 2006-12-20

**Authors:** Ichizo Morita, Haruo Nakagaki, Atsushi Toyama, Matsumi Hayashi, Miho Shimozato, Tsuyoshi Watanabe, Shimpei Tohmatsu, Junko Igo, Aubrey Sheiham

**Affiliations:** 1 Department of Preventive Dentistry and Dental Public Health, School of Dentistry, Aichi-Gakuin University, Nagoya, Japan; 2 Department of Epidemiology and Public Health, University College London, London, UK; 3 Tobishima Village, Ama-Gun, Japan; 4 Ama Dental Association, Ama, Japan; 5 Aichi Dental Association, Nagoya, Japan; 6 Aichi Prefecture, Nagoya, Japan

## Abstract

**Background:**

The aim of this study was to determine which behavioral factors to include in guidelines for the Japanese public to achieve an acceptable level of oral healthiness. The objective was to determine the relationship between oral health related behaviors and symptoms related to oral disease and tooth loss in a Japanese adult community.

**Methods:**

Oral health status and lifestyle were investigated in 777 people aged 20 years and older (390 men and 387 women). Subjects were asked to complete a postal questionnaire concerning past diet and lifestyle. The completed questionnaires were collected when they had health examinations. The 15 questions included their preference for sweets, how many between-meal snacks they usually had per day, smoking and drinking habits, presence of oral symptoms, and attitudes towards dental visits. Participants were asked about their behaviors at different stages of their life. The oral health examinations included examination of the oral cavity and teeth performed by dentists using WHO criteria. Odds ratios were calculated for all subjects, all 10 year age groups, and for subjects 30 years or older, 40 years or older, 50 years or older, and 60 years or older.

**Results:**

Frequency of tooth brushing (OR = 3.98), having your own toothbrush (OR = 2.11), smoking (OR = 2.71) and bleeding gums (OR = 2.03) were significantly associated with number of retained teeth in males. Frequency of between-meal snacks was strongly associated with number of retained teeth in females (OR = 4.67). Having some hobbies (OR = 2.97), having a family dentist (OR = 2.34) and consulting a dentist as soon as symptoms occurred (OR = 1.74) were significantly associated with number of retained teeth in females. Factors that were significantly associated with tooth loss in both males and females included alcohol consumption (OR = 11.96, males, OR = 3.83, females), swollen gums (OR = 1.93, males, OR = 3.04, females) and toothache (OR = 3.39, males, OR = 3.52, females).

**Conclusion:**

Behavioral factors that were associated with tooth retention were frequency of eating snacks between meals, tooth brushing frequency, having one's own toothbrush, smoking and drinking habits, having hobbies, having a family dentist and when they had dental treatment. Clinical factors included bleeding gums, swollen gums, and toothache.

## Background

The 8020 Campaign was started in 1989 to promote oral health in Japan [1,2]. The objective of the campaign is to encourage people to retain 20 or more natural teeth at 80 years of age. That acceptable level of oral health was based on recommendations by Chief Dental Officers of Northern European countries [3]. Having 20 teeth at 80 years was considered sufficient for people to eat and enjoy meals with a wide range of foods. The dental 80:20 concept is supported by numerous studies in the Netherlands [4,5], United Kingdom [6], USA [7] and Japan [8]. Kayser [4,5] reported that at least 12 front teeth and 8 premolars are necessary for satisfactory biting and chewing. Similar conclusions were drawn in Japan by Goto et al [9]. Namely that retaining more than 20 teeth is adequate for mastication. Another Japanese study found that people retaining 20 or more teeth could eat most types of Japanese foods. Based mainly on Kayser's findings, a World Health Organization [3] workshop determining acceptable levels of oral health adopted as a goal for oral health 'the retention throughout life of a functional, aesthetic, natural dentition of not less than 20 teeth (shortened dental arch) and not requiring recourse to a prosthesis'. So there is considerable evidence that 80:20 is a reasonable and acceptable oral health goal.

In the year 2000, the Japanese Ministry of Health, Labour and Welfare began a national health plan, "Healthy Japan 21", focusing on health promotion and increasing of disability-adjusted life expectancy. The plan stipulated that local governments are responsible for establishing and executing their own plans [10], which includes the promotion of better oral health to achieve the goals of Healthy Japan 21. Under the plan the role of local government is to enable people to have more control over their health and enjoy healthier lifestyles by creating health supporting environments [11]. The contribution of the dental profession towards that objective is to develop a practical appropriate and acceptable set of guidelines to help people maintain good oral health and prevent tooth loss sothat they can retain at least 20 teeth for their lifetimes. Evidence of which behaviors contribute to retaining 20 or more teeth for a lifetime are required for developing such evidence based guidelines.

To promote the health and oral health of residents in communities, data on factors affecting oral health obtained in previous studies should be expressed in easily understood terms sothat they can be understood by policy makers and lay people. A person might find lifestyle changes difficult to make if others in their group do not try to make changes as well [12]. For these reasons, residents require guidelines that can be widely applied. Such guidelines should be attractive to people and interest local industries such as food companies and companies making oral health related products, such as toothbrushes and other aids. To promote the well-being of residents, local authorities must formulate and implement health-promoting programs specifically designed for the community to enable people to develop personal skills and to create supportive environments [11].

Although people in Japan are aware of some of the behaviors related to oral disease they need some more practical guidelines to help them to improve their oral health related behaviors to maintain good oral health. The guidelines should be based on sound evidence on what factors affect tooth loss and tooth retention.

Dental and oral health are affected by diet and certain aspects of lifestyle [13-24]. Burt et al [13] concluded "that total tooth loss was a social-behavioral issue as much as it is disease related". They reported that socio-behavioral factors were less clearly related to partial tooth loss in dentate persons. In a later national study, Eklund and Burt [16] reported there were associations between total tooth loss and low income, education, perceived poor oral health, smoking and negative health behavior. Their findings on the importance of socio-behavioral factors for tooth loss were confirmed by Gilbert et al [20] who found that those 65 year old Floridians with less positive attitudes to dentists, and who practiced dental hygiene less frequently and were smokers had lost more tooth. Similar findings, namely that brushing teeth infrequently and being a smoker affected tooth loss, were reported by a number of authors [19,21,24]. The importance of smoking and heavy drinking as contributing factors to tooth loss in older people was also highlighted by Klein et al [22]. Some researchers reported socio-economic inequalities were related tooth loss [24-27]. The factors mentioned above may not be important in all populations. For example, in a study of two longitudinal cohorts, the Baltimore Longitudinal Study of Aging (BLSA) and the V.A. Dental Longitudinal Study (VALS), Copeland et al [23] found that in the two US adults studies the risk factors for tooth loss differed. By analyzing which factors affected tooth loss policy makers can develop guidelines for younger cohorts. All but one of the studies mentioned above were done outside Japan. Because the factors affecting tooth retention in Japan may differ from those in other countries a study was planned to establish which behavioral factors to include in guidelines that should promote an acceptable level of oral healthiness, having 20 teeth at 80 years in Japanese adults, a study was planned with the objective of determining the relationship of specific oral health related behaviors and symptoms of oral disease and number of retained teeth in a typical Japanese community.

## Methods

### Subjects

Tobishima was chosen as the study site because it is typical of medium sized village communities in Japan. All 3,619 residents aged 20 years and over living in Tobishima were contacted and asked to participate in the annual health check recommended by the Ministry of Health, Labour and Welfare. Some of them chose to be examined at their workplace. They were not included in this general and dental health study for logistical reasons. That explains the low response rate of 21.5% for this general and dental health survey. Nevertheless, 390 men and 387 women took part in the dental survey in 1998. The age distribution of the study group was similar to that of all residents aged 30 to 60 years. People in their 20s and 70 years and older were under-represented in the study (Table 1).

**Table 1:** Age and sex distribution of the Tobishima study participants

	Study participants				Whole village^a^			
Age (years)	Male	Female	Total	(%)	Male	Female	Total	(%)

20–29	7	8	15	1.9	339	258	597	16.5
30–39	49	80	129	16.6	233	264	497	13.7
40–49	107	117	224	28.8	372	347	719	19.9
50–59	94	85	179	23.0	357	298	655	18.1
60–69	97	79	176	22.7	265	284	549	15.2
over 70	36	18	54	6.9	229	373	602	16.6

Total	390	387	777	100.0	1795	1824	3619	100.0

a) In July 1998

The study was reviewed and approved by the Ethical Committee of Aichi-Gakuin University (Reference number is 12).

### Questionnaire and examinations

Subjects were asked to complete questionnaires concerning past diet and lifestyle. The questionnaire was mailed to each participant and the completed questionnaires containing 40 questions were collected when they had their health examinations. Of the 40 questions, those relevant to tooth retention were selected and used for this study. Fifteen questions were used in this study to investigate lifestyle and symptoms related to the teeth and mouth. These questions were chosen from among more than 40 that had been used in previous studies of elderly people in Japan [1,2]. The 15 questions included questions on whether the subjects had a preference for sweet foods including confectionary, how many between-meal snacks they had per day, smoking and drinking habits, the presence of symptoms related to the teeth and mouth, and attitudes towards dental check-ups (Table 2). Participants were asked about their behaviors at different stages of their life; when they were in elementary school and junior high school and when they were in their 20s, 30s, 40s, and 50s. The questions and answer options are presented in Table 2.

**Table 2:** Questions and responses at different stages of life

	Questions	Responses at different periods of the life course	Options
Q1	Preferred intake of sweet food	ES, JHS, 20, 30, 40, 50	Yes/Moderate, No
Q2	Try not to eat sweets	ES, JHS, 20, 30, 40, 50	Yes, Moderate/No
Q3	Frequent between-meal snacks	ES, JHS, 20, 30, 40, 50	Always/Sometimes, Never
Q4	Frequency of tooth brushing	ES, JHS, 20, 30, 40, 50	2 or more times/1 or fewer times
Q5	Have your own tooth brush	20, 30, 40, 50	Yes/No
Q6	Smoking	20, 30, 40, 50	Yes/No, Quit
Q7	Alcohol	ES, JHS, 20, 30, 40, 50	Yes/No
Q8	You have some hobbies	ES, JHS, 20, 30, 40, 50	Yes/No
Q9	At least one dental clinic near your house	ES, JHS, 20, 30, 40, 50	Yes/No
Q10	You have a family dentist	ES, JHS, 20, 30, 40, 50	Yes/No
Q11	Consult a dentist as soon as dental symptoms appear	ES, JHS, 20, 30, 40, 50	Yes/No
Q12	Gum bleeding	20, 30, 40, 50	Frequently, Occasionally/Very seldom
Q13	Gum swelling	20, 30, 40, 50	Frequently, Occasionally/Very seldom
Q14	Toothache	20, 30, 40, 50	Frequently, Occasionally/Very seldom
Q15	Scaling	20, 30, 40, 50	Frequently, Occasionally/Very seldom

ES: When a elementary school student

JHS: When a junior high school student

20, 30, 40, 50: When you are/were 20/30/40/50 years old

The oral health examination included clinical examination of the oral cavity and teeth performed by dentists using adequate lighting, a dental mirror, and probe according to pre-established criteria. The dental health examination was conducted at the health center at Tobishima. For each subject the number of retained teeth, excluding wisdom teeth, was counted according to a modified World Health Organization checklist [28]. Other dental data, such as DMF and CPI were also recorded but not used in the present analysis.

### Statistical analysis

**Figure 1:**
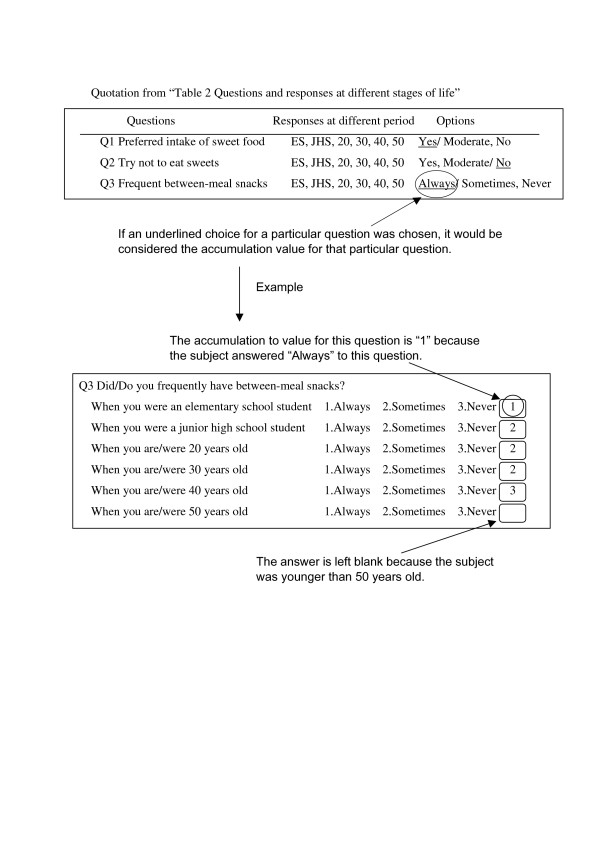
Process of calculating accumulation values.

All subjects were examined to determine whether they had more or less than the average number of teeth for their respective groups. Questions that were rarely chosen were combined with other relevant questions. When questions had three or more choices, they were further combined to only 2 choices. The choice of answer that was underlined was chosen as the answer for a particular question (Table 2) (Figure 1).

Cross tabulation and odds ratio were used to assess bivariate relationships. An odds ratio of 1 was assigned to the answers in parentheses (Table 4), and the risks of tooth loss associated with other choices were calculated. Odds ratios were obtained for all subjects, for all 10 year age groups, and for subjects 30 years or older, 40 years or older, 50 years or older, and 60 years or older. Odds ratios were also determined separately for men and women. Analyses were undertaken by using SPSS 11.0J for Windows.

**Table 4:** Oral health related questions that had statistically significant odds ratios of being related to cut off groupings of retained teeth.

		Items														
	Age	Q1	Q2	Q3	Q4	Q5	Q6	Q7	Q8	Q9	Q10	Q11	Q12	Q13	Q14	Q15

Male	All ages	-	-	-	1.55	2.11	-	-	-	-	-	-	2.03	2.27	2.28	-
	30–39	-	-	-	-	-	-	-	-	-	-	-	-	-	-	-
	40–49	-	-	-	-	-	-	-	-	-	-	-	-	-	-	-
	50–59	-	-	-	3.98	-	-	11.96	-	-	-	-	-	-	-	-
	60–69	-	-	-	-	-	-	-	-	-	-	-	-	-	3.39	-
	70–79	-	-	-	-	-	-	-	-	-	-	-	-	-	-	-
	Over 30	-	-	-	1.56	2.07	-	-	-	-	-	-	2.02	2.19	2.18	-
	Over 40	-	-	-	1.93	1.97	-	-	-	-	-	-	2.02	2.16	2.00	-
	Over 50	-	-	-	3.25	-	2.71	-	-	-	-	-	1.83	2.13	-	-
	Over 60	-	-	-	2.41	-	-	-	-	-	-	-	-	-	2.38	-

Female	All ages	-	-	1.81	-	-	-	-	-	-	-	1.72	-	2.04	-	-
	30–39	-	-	-	-	-	-	-	-	-	-	-	-	-	-	-
	40–49	-	-	2.24	-	-	-	3.83	-	-	2.34	-	-	3.04	3.52	-
	50–59	-	-	-	-	-	-	-	2.97	-	-	-	-	-	-	-
	60–69	-	-	4.67	-	-	-	-	-	-	-	-	-	-	-	-
	70–79	-	-	-	-	-	-	-	-	-	-	-	-	-	-	-
	Over 30	-	-	1.81	-	-	-	-	-	-	-	1.74	-	1.93	-	-
	Over 40	-	-	2.25	-	-	-	-	-	-	-	-	-	2.34	-	-
	Over 50	-	-	2.40	-	-	-	-	-	-	-	-	-	2.02	-	-
	Over 60	-	-	4.08	-	-	-	-	-	-	-	-	-	-	-	-

Only significant odds ratios at 95% CI is shown.																

Q1	Preferred intake of sweet food								Yes/(Moderate, No)							
Q2	Try not to eat sweet								(Yes, Moderate)/No							
Q3	Frequent between-meal snacks								Always/(Sometimes, Never)							
Q4	Frequency of tooth brushing								2 or more times/(1 or fewer times)							
Q5	Have your own tooth brush								(Yes)/No							
Q6	Smoking								Yes/(No, Quit)							
Q7	Alcohol								Yes/(No)							
Q8	You have some hobbies								(Yes)/No							
Q9	At least one dental clinic near your house								(Yes)/No							
Q10	You have a family dentist								(Yes)/No							
Q11	Consult a dentist as soon as symptoms appear								(Yes)/No							
Q12	Gum bleeding								Frequently, Occasionally/(very seldom)							
Q13	Gums swelling								Frequently, Occasionally/(very seldom)							
Q14	Toothache								Frequently, Occasionally/(very seldom)							
Q15	Scaling								(Frequently, Occasionally)/very seldom							

/: Options used in statistical analysis

-: No Significance:

The items in brackets is reference is reference (odds ratio = 1).

The outcome variable was coded 1: at or above cut-off point, 0: below cut-off point.

The cut-off points varied according to age and sex group (see Table 3).

## Results

### Number of retained teeth in each age group

Men and women aged 20 to 29 and 30 to 39 years retained an average 28 teeth. The 40 to 49 years age groups of both men and women retained 27 teeth, and the 50 to 59 groups of both men and women retained on average 24 teeth. In the 60 to 69 group men retained 18, and women 21 teeth. In the 70 years and older group, men retained 14 and women, 16 teeth. One third of all subjects (133 men and 132 women) had fewer teeth than the average for their respective age groups (Table 3).

**Table 3:** Cut off points and number of participants below and above the cut-off points

		Males				Females	
Age (in years)	Cut off point^a^	Below Cut off point^b^	Above Cut off point^b^		Cut off point^a^	Below Cut off point^b^	Above Cut off point^b^

20–29	28	1	6		28	0	8
30–39	28	13	36		28	20	60
40–49	27	38	69		27	44	73
50–59	24	26	68		24	32	53
60–69	18	38	59		21	28	51
over 70	14	17	19		16	8	10

Total		133	257			132	255

a) Average number of teeth

b) Number of participants

### Relationship between the number of retained teeth and lifestyle and clinical factors

Four of the 15 questions in the questionnaire were not significantly related to tooth retention in any of the age groups (Table 4). They were, preferred intake of sweet foods, try not to eat sweets, at least one dental clinic near your house and having had dental scaling. The remaining 11 items had a significant relationship in at least one age group.

Frequency of tooth brushing (OR = 3.98, 95%CI: 1.42–11.14 at 50–59 year old group), having own toothbrush (OR = 2.11, 95%CI: 1.11–4.02 at all ages)", smoking (OR = 2.71, 95%CI: 1.07–6.89 at over 50 years old groups) and bleeding gums (OR = 2.03, 95%CI: 1.25–3.30 at all ages) were significantly associated with number of retained teeth in males.

Frequency of between-meals snacks was associated with number of retained teeth in females. The relationship was very strong in 60 to 69 year old females (OR = 4.67, 95%CI: 1.66–13.11). In addition having some hobbies (OR = 2.97, 95%CI: 1.13–7.78 in the 50–59 year group), having a family dentist (OR = 2.34, 95%CI: 1.03–5.34 in the 40–49 year group) and consulting a dentist as soon as symptoms occur (OR = 1.74, 95%CI: 1.07–2.84 in the over 30 year old groups) were significantly associated with number of retained teeth in females.

Factors that were significantly associated with tooth loss in both males and females included alcohol consumption (OR = 11.96, 95%CI: 1.52–94.03 in 50 to 59 year male group, OR = 3.83, 95%CI: 1.08–13.60 at 40–49 year female group, swollen gums (OR = 1.93, 95%CI: 1.22–3.05 in over 30 year old male group, OR = 3.04, 95%CI 1.28–7.22: at 40–49 year female group) and toothache (OR = 3.39, 95%CI: 1.15–10.01 at 60 to 69 year male group, OR = 3.52, 95%CI: 1.11–11.15 at 40–49 year female group).

## Discussion

The aims and objectives of this study were to establish which behavioral factors to include in guidelines that should promote oral healthiness for a lifetime, here considered to be retaining at least 20 teeth at age 80 years. The outcome measure was tooth loss, the obverse of tooth retention. Tooth loss is mainly caused by dental caries and periodontal diseases [29]. Their onset and development are influenced by the accumulation of many determinants that are present for a long time. Therefore it is important to use information from across the whole life course [30,31]. The results from this study reflect past and present behaviors and oral symptoms because the cumulative values for each question were considered to be important.

In this retrospective study, we examined the history of each subject's behaviors and subjective conditions of the oral cavity that may have influenced their dentally related behaviors. Therefore, for example, a 70-year-old man was asked to recall conditions and behaviors when he was an elementary school student 60 years ago. Views on the reliability of memories differ. Some reported that original dietary reports and retrospective reports after 3 to 14 years have good correlation coefficients of 0.5 to 0.7 [32-34]. Berney et al. [35] reported that after a period of 50 years people recalled socio-demographic information remarkably accurately. Questions in our study were mainly about lifestyle, and we assume that the retrospective recall of their earlier lifestyle were at least as reliable as retrospective dietary reports.

Our results on behavioral factors affecting tooth retention were similar to those of other workers excepting that the importance of between meal snacks, a well established cause of dental caries, was the most significant factor linked to tooth loss in our Japanese population. This study found that in order of importance, frequency of between-meal snacks, alcohol consumption, smoking, frequency of tooth brushing, having some hobbies, having a family dentist and consulting a dentist when dental symptoms such as bleeding gums or toothache occurred, were significantly associated with number of retained teeth.

Kressin et al [19] reported that adherence to American Dental Association recommendations that individuals brushing twice and flossing at least once a day for preventive care would lead to better oral health. Drake et al [17] and Hunt et al [36] using data from follow-up studies over 18 months and 3 years, respectively, found that elderly white Americans whose teeth were sensitive to cold or hot foods and who had pain in the oral cavity were more likely to lose their teeth and Burt et al [13] reported that having gingivitis was related to tooth loss (OR, 2.4 [95%CI: 1.2–5.2]). In addition, Eklund et al [16] reported that poor general and oral health and the absence of regular dental visits were strongly associated with the risk of tooth loss. They have also reported that tooth loss was correlated with higher periodontal disease scores, perceived poor dental health, and a history of smoking in younger people. Stress is believed to contribute to various diseases and is reportedly associated with tooth loss [37]. In addition to some of the questions by the abovementioned researchers our questionnaire included a question on hobbies to determine whether having a hobby may reduce dental disease and tooth loss. Our research does not clarify whether having a hobby will change the risk of tooth loss at an individual level. It may point to the importance of flexibility in daily routines and/or stress. We found that persons with hobbies lost fewer teeth than those without a hobby. This finding may be due to co-variance. People with more time for hobbies may be higher socioeconomic status. They have more flexibility in their daily activities and routines and that influences their mouth cleaning behaviors and periodontal health [38,39]. In addition, having a hobby may reduce stress, and stress affects periodontal status [40].

The findings from this study have implications for developing guidelines on retaining sufficient teeth to function normally in older age. The finding that diet, alcohol consumption, smoking, oral cleanliness, having hobbies and using dental services sensibly fits well with WHO guidelines on the prevention of chronic diseases and the common risk factor approach [41] and can therefore readily be incorporated with general guidelines for the Japan national health plan, "Healthy Japan 21". The key concept underlying the integrated Common Risk Factor Approach is that promoting general health by controlling a small number of risk factors, may have a major impact on a large number of diseases at a lower cost and greater efficiency and effectiveness than disease specific approaches [42,43]. Savings may be made by coordinating the work done by various specialist groups and organizations. Decision-makers and individuals will be more readily influenced by measures directed at preventing heart diseases, obesity, stroke, cancers, diabetes as well as dental diseases than if disease-specific recommendations are made.

The findings from this study will be used in the development of a self-administered checklist. The checklist will be used to assess present behaviors and symptoms and suggest using the guidelines for promoting 'oral healthiness'.

## Conclusion

The objective of this study was to assess the relationship of oral health related behaviors and symptoms related to oral disease and tooth loss sothat they can be included in guidelines for the public about how to maintain 'oral healthiness' for their lifetimes. Although our questions did not assess their oral health related quality of life, but concentrated on tooth loss, the overall findings suggest that the factors that were associated with tooth retention were frequency of between-meal snacks, alcohol consumption, smoking, frequency of tooth brushing, having some hobbies, having a family dentist and consulting a dentist when dental symptoms such as bleeding gums or toothache occurred, were significantly associated with number of retained teeth. Clinical factors included bleeding gums, swollen gums, and toothache.

## Competing interests

The author(s) declare that they have no competing interests.

## Authors' contributions

IM: contributed to study design, carried out the study, data collection, statistical analysis and manuscript writing. HN: conceived of the study, participated in study design, and manuscript writing. AT: contributed to statistical analysis. MH, MS, TW, ST and JI: contributed to data collection. AS: participated in study design, writing and reviewing manuscript.

## Pre-publication history

The pre-publication history for this paper can be accessed here:


